# Anti-Tumorigenic Activities of IL-33: A Mechanistic Insight

**DOI:** 10.3389/fimmu.2020.571593

**Published:** 2020-11-30

**Authors:** Sara Andreone, Adriana Rosa Gambardella, Jacopo Mancini, Stefania Loffredo, Simone Marcella, Valentina La Sorsa, Gilda Varricchi, Giovanna Schiavoni, Fabrizio Mattei

**Affiliations:** ^1^Department of Oncology and Molecular Medicine, Istituto Superiore di Sanità, Rome, Italy; ^2^Department of Translational Medical Sciences and Center for Basic and Clinical Immunology Research (CISI), University of Naples Federico II, Naples, Italy; ^3^Institute of Experimental Endocrinology and Oncology “G. Salvatore”, National Research Council (CNR), Naples, Italy; ^4^Research Coordination and Support Service, CoRI, Istituto Superiore di Sanità, Rome, Italy

**Keywords:** IL-33, tumor microenvironment, tumor immunity, eosinophils, ILC2, CD8 T cells, immune checkpoints, basophils

## Abstract

Interleukin-33 (IL-33) is an epithelial-derived cytokine that can be released upon tissue damage, stress, or infection, acting as an alarmin for the immune system. IL-33 has long been studied in the context of Th2-related immunopathologies, such as allergic diseases and parasitic infections. However, its capacity to stimulate also Th1-type of immune responses is now well established. IL-33 binds to its specific receptor ST2 expressed by most immune cell populations, modulating a variety of responses. In cancer immunity, IL-33 can display both pro-tumoral and anti-tumoral functions, depending on the specific microenvironment. Recent findings indicate that IL-33 can effectively stimulate immune effector cells (NK and CD8^+^ T cells), eosinophils, basophils and type 2 innate lymphoid cells (ILC2) promoting direct and indirect anti-tumoral activities. In this review, we summarize the most recent advances on anti-tumor immune mechanisms operated by IL-33, including the modulation of immune checkpoint molecules, with the aim to understand its potential as a therapeutic target in cancer.

## Introduction

Interleukin-33 (IL-33) was initially described by JP Girard’s group as a nuclear factor from high endothelial venules (NF-HEV) (1). It was later rediscovered, by a computational sequence search, as an IL-1 family member (2). Although initially defined as an immune component of Th2 response, its pleiotropic contribution to the immune response has now emerged. Hence, IL-33 has been involved in different immune processes, such as inflammatory diseases, allergies, infections and cancer (3). IL-33 is expressed as a nuclear factor by different types of cells, such as endothelial cells, fibroblasts, epithelial cells and other stromal cells (4). In the tumor microenvironment (TME), these cells, together with tumor cells and some immune infiltrating cells, are an important source of IL-33 (5, 6). Like high-mobility group box 1 protein (HMGB1), IL-33 is released outside the cell after stress or damage and acts as an alarmin that activates the immune response (7). Two different isoforms of IL-33 have been described: the IL-33 full-length form (IL-33 FL) and the IL-33 mature form (8, 9). Several inflammatory proteases, mostly derived from neutrophils and mast cells, can process IL-33 FL into the mature form, endowed with superior (10- to 30-fold) bioactivity (4). Since both neutrophils (10) and mast cells (11) are recruited in the TME, these proteases may be abundantly present thus amplifying IL-33 activity. On the other hand, the pro-inflammatory action of IL-33 may be controlled by oxidation (12) or proteolytic cleavage by apoptotic caspases (13), leading to IL-33 inactivation. Therefore, the balance between different proteases as well as the nature of tumor cell death (necrotic *vs* apoptotic) may dictate the activity of IL-33 within the TME.

IL-33 binds to a heterodimer formed by its primary receptor ST2 and the co-receptor IL-1 receptor accessory protein (IL1RAP). This activates a signal cascade through MyD88-IRAK-dependent pathway, and leads to NF-κB, c-Jun N-terminal kinase (JNK) and mitogen-activated protein kinase (MAPK) activation (2), which results in the release of a plethora of soluble mediators by different immune cells (14). IL1RAP is constitutively expressed at low levels by virtually all cells, including immune cells (15). ST2 is expressed primarily by cells involved in Th2 response, such as Th2 cells, eosinophils, basophils, mast cells, a subset of regulatory T cells (Treg) and type 2 innate lymphoid cells (ILC2), but also by Th1 cells, CD8^+^ T cells, NK cells, macrophages, neutrophils, dendritic cells (DC) and B cells (16, 17). A soluble form of ST2 (sST2) exists as a decoy receptor that prevents IL-33 binding to the transmembrane receptor (18). Tumor, epithelial and immune cells express sST2 at various levels, which may contribute to regulate the availability of IL-33 in the TME (19).

The IL-33/ST2 axis has a controversial role in cancer immunity, since both pro- and anti-tumoral functions have been reported. This dichotomy seems to depend on multiple factors, such as the composition of the TME and tissue of tumor origin, and has been reviewed recently (16). In this mini review, we will focus on the anti-tumor effects of IL-33/ST2, with emphasis on the most recent advances on immune mechanisms and their potential exploitation for future therapeutic options.

## IL-33 Promotes the Effector Functions of CD8^+^ T and NK Cells

Several studies demonstrated that IL-33 expression positively correlates with CD8^+^ T and NK cell recruitment and activation in the TME. Transgenic expression of IL-33 in B16 or 4T1 tumor cells (20) or in the host (21), as well as exogenous administration of the recombinant protein (22) induce the recruitment of activated (IFN-*γ*^+^ CD107^+^) CD8^+^ T and NK cells in the TME, which inhibited tumor growth in mice. In a breast cancer model, IL-33 induced the recruitment and activation of NK cells to the lung that prevented pulmonary metastasis onset (23). IL-33 can increase the cytotoxicity of CD8^+^ T cells and NK cells also *in vitro*, indicating a direct action (21). Both FL and mature IL-33 isoforms acted as adjuvants in an HPV DNA vaccination model promoting antigen-specific CD8^+^ T cell expansion and degranulation that resulted in regression of established TC-1 lung tumors (24). Although these findings point to a similar biological activity of FL and mature IL-33 isoforms, the possibility that FL IL-33 is converted into the mature form once released in the TME and exposed to local proteases cannot be excluded (9, 24).

Mechanistically, the ability of IL-33 to induce tumor-reactive IFN-*γ*^+^ CD107^+^ CD8^+^ T and NK cells was recently shown to be dependent on MyD88 signaling in a mouse model of Lewis lung carcinoma (25). Furthermore, the IFN-inducing DNA sensor STING promoted tumor cytotoxicity by stimulating some chemokines (CXCL10 and CCL5) and IL-33, which participated in NK cell infiltration and activation in a mouse model of NK-sensitive melanoma (26). These studies reveal a possible link between IL-33 and IFN-related response in cancer immunity, as already reported in IgG4-related autoimmune diseases (27).

The role of endogenous IL-33 in mediating CD8^+^ T cell-dependent antitumor responses was also demonstrated. In murine hepatocellular carcinoma, tumor-derived IL-33 promoted the expansion of IFN-*γ*^+^ CD4^+^ and CD8^+^ T cells, increased CTL cytotoxicity and inhibited tumor growth (28). Induction of IL-33 production by stromal cells following LCMV-based vector immunotherapy elicited protective anti-tumor CD8^+^ T cell effector responses (29). In a colon carcinoma model, endogenous IL-33 promoted IFN-*γ* expression by both CD4^+^ and CD8^+^ T cells, increased CD8^+^ T cell infiltration over Treg cells and augmented CD8^+^ T cell-mediated antitumor responses (30). These observations imply that endogenous levels of IL-33 by tumor and stromal cells may support cancer immune surveillance by CD8^+^ T cells.

IL-33 can promote the effector functions of CD8^+^ T cells also through stimulation of DC. IL-33 administration in tumor-bearing mice activated DC and increased Ag cross-presentation to CD8^+^ T cells in melanoma (31) and acute myeloid leukemia (AML) models (32). One group reported that IL-33-stimulated DC expand a population of cytotoxic IL-9 producing CD8^+^ T cells, termed Tc9, endowed with potent anti-tumor activity in melanoma-bearing mice (33). The relevance of Tc9 cells in human cancers is still unclear.

Notably, IL-33 is implicated in the differentiation of T cells into tissue-resident memory T (T_RM_) cells, a recently identified CD8^+^ T cell population found in various human cancers and correlating with favorable outcome (34). These cells express the integrins CD103 and CD49a and the C-type lectin CD69, and are characterized by *in situ* proliferation, location and persistence in close contact with malignant cells, *via* binding of CD103 to tumor E-cadherin (35). Whether and how IL-33 can affect T_RM_ in cancer warrants investigation.

## Modulation of CD4^+^ T Cell Functions by IL-33 in the TME

Both conventional and regulatory CD4^+^ T cells are direct targets of IL-33. IL-33 can promote the recruitment and the immunosuppressive functions of Treg cells expressing ST2, favoring tumor growth and immunoevasion (36–39). On the other hand, IL-33 can activate conventional Th cells, inducing their phenotypic polarization, clonal expansion, and cytokine production (40). IL-33 preferentially promotes Th2 response, which is classically believed to contrast tumor immunity, although its role appears ambivalent (41). Under some conditions, such as in the presence of IL-12, IL-33 can induce Th1 responses (42, 43). In an HPV-associated mouse tumor model, IL-33 promoted IFN-*γ* and TNF-α production by antigen-specific CD4^+^ T cells (24). Similarly, IL-33 was reported to amplify IFN-*γ*^+^ CD4^+^ T cells in mouse models of hepatocellular (28) and colon carcinoma (30, 44). These data demonstrated that IL-33 has the capacity to promote Th1-mediated anti-tumor response.

Lastly, IL-33 also promotes the differentiation of IL-9-producing Th cells (45), which exert potent antitumor activity in certain solid cancers, such as melanoma (46). Therefore, IL-33 can differently regulate CD4^+^ T cell polarization and function in the TME. A comprehensive analysis of cytokine profiles activated by IL-33 in various cancers may help clarify the CD4^+^ T cell subsets (including Treg) targeted by IL-33 in relation to the specific TME and anti-tumor response elicited.

## IL-33 activates eosinophils, basophils, and mast cells

Eosinophils infiltrate most human and experimental cancers where they play diverse roles (47). Migration to the TME can be mediated by eotaxins (eotaxin-1/CCL11, eotaxin-2/CCL24, eotaxin-3/CCL26) that bind the CCR3 receptor highly expressed on eosinophils (47, 48) and by alarmins (i.e., HMGB1 and IL-33) released from dying tumor cells (22, 49). Whereas HMGB1 is a direct chemoattractant for eosinophils (50), IL-33 appears to recruit eosinophils only indirectly, *via* stimulation of tumor-released chemokines, such as CCL24 (51, 52), or through the activation of IL-5 producing ILC2 (53–55) and mast cells (56).

Several studies demonstrated the role of eosinophils in mediating the anti-tumoral activities of IL-33. Injection (22) or tumor expression (57) of IL-33 in melanoma-bearing mice inhibited tumor growth and this effect was abolished upon eosinophil depletion by injections of anti-Siglec-F mAb. In models of transplantable and colitis-associated colorectal cancer, tumor growth reduction induced by IL-33 was abrogated in eosinophil-deficient ΔdblGATA-1 mice, but was restored by adoptive transfer of eosinophils activated with IL-33 *ex vivo* (52). Mechanistically, eosinophils can exert anti-tumor activity partly by promoting the recruitment of CD8 T cells (22, 58). In fact, eosinophils are an important source of chemokines (CCL5, CXCL9, CXCL10) that attract CD8^+^ T cells in TME (58) and can be up-regulated by administration of IL-33 (22). Moreover, eosinophils can exert direct tumor cytotoxicity (22, 51, 52). In a model of pulmonary melanoma metastasis, eosinophil depletion caused the inhibition of metastasis formation in mice receiving IL-33, without apparent involvement of cytotoxic CD8^+^ T cells, thus suggesting an active role of eosinophils in the lung (22). In fact, IL-33 can directly activate human (59, 60) and mouse (52, 61) eosinophils by up-regulating activation markers (i.e. CD69), adhesion molecules (i.e., ICAM-1 and CD11b/CD18), and the degranulation markers CD63 and CD107a, resulting in the killing of several tumor cell types (51, 52, 62, 63). Once activated with IL-33, these granulocytes exert tumor cytotoxic functions through contact-dependent degranulation, involving polarization of eosinophilic eﬀector proteins (eosinophil cationic protein, eosinophil peroxidase, and granzyme B) and convergence of lytic granules to the immunological synapses (51). This study provides the first evidence that eosinophils during degranulation employ a mechanism similar to that used by NK cells (64).

IL-33 is able to activate murine and human basophils, increasing histamine and cytokine production *in vitro* and promoting their expansion *in vivo* (16, 65–67). IL-33 can synergize with IL-3 to induce IL-9 production in human basophils (68), which may support tumor immunity (69). In human basophils, IL-33 alone does not directly induce degranulation but can enhance IL-3- and anti-IgE-mediated degranulation (67, 70). Recently, our group reported that mouse basophils stimulated with IL-33 up-regulate the expression of granzyme B and of the degranulation marker CD63 and induce melanoma cell killing *in vitro* (71). Although the role of basophils in cancer immunity is still unclear (72), this latter observation may broaden the spectrum of immune effector cells that can be activated by IL-33 within the TME.

Mast cells infiltrate several types of experimental and human tumors (56, 73). IL-33 activates human mast cells to release several cytokines (74) and enhances immune complex-triggered activation of human mast cells (75). Furthermore, IL-33 increases the expression of ICAM-1 (76) and MHC-II (77), and promotes the survival (78) and degranulation (79) of murine mast cells. However, due to the wide range of mediators they release, it is difficult to define the pro- or anti-tumorigenic activity of mast cells (11).

## IL-33 as an enhancer of anti-Tumor activities of ILC2

ILC2 constitutively express ST2 and respond directly to IL-33, which is necessary for their expansion, recruitment and activation (80, 81). Two distinct subsets of ILC2 have been described: resident natural ILC2 and inflammatory ILC2, which can be induced upon IL-33 stimulation (81). High numbers of ILC2 can be found in many IL-33-enriched tumors, although their role in cancer immunity remains controversial (82). Ikutani et al. first described an anti-tumoral role of ILC2 in a mouse model of melanoma. In this study, systemic IL-33 injections expanded IL-5-producing ILC2 that induced eosinophil recruitment, which were critical to suppress pulmonary metastases (54). In another study, inoculation of IL-33-expressing EL4, CT26 or B16.F10 tumor cells induced MyD88-dependent intratumoral expansion of ILC2 in mice that were indispensable for IL-33-mediated antitumor activity independently of eosinophils (83). In this model, ILC2 exerted anti-tumoral activity through production of CXCL1 and CXCL2. Binding of these chemokines to tumor cell-expressed CXCR2, which was sustained by the hypoxic TME created by IL-33, resulted in tumor cell apoptosis. This study first demonstrated that activated ILC2 can be cytotoxic for tumor cells.

A recent study on the B16.F10 melanoma model showed that TME acidification caused by lactic acid (LA) produced by the tumor impaired ILC2 survival and function (55). This prevented tumor infiltration of ILC2 and resulted in rapid tumor growth. Accordingly, gene expression analysis in human cutaneous melanomas revealed an inverse correlation between lactate dehydrogenase A (LDHA, the enzyme responsible for LA production) and markers associated with ILC2. *In vivo* interference with LDHA in B16.F10 tumors or administration of IL-33 to tumor-bearing mice increased the number of intratumoral ILC2 and restored ILC2 ability to contrast tumor progression. IL-33 also induced an increase in the number of tumor infiltrating eosinophils. This study reveals an anti-tumorigenic role of IL-33/ILC2/eosinophils axis controlled by glucose metabolism.

Moral and co-workers reported that ILC2 infiltrate human and mouse pancreatic ductal adenocarcinomas (PDAC) (84). High frequencies of tumor-infiltrating ILC2 (TILC2) were found in “hot” tumors (enriched in CD8^+^ T cells), and correlated with better survival and high expression of IL-33. By comparing the effects of IL-33 deficiency (or exogenous administration) on orthotopic PDAC and heterotopic skin tumor growth, the authors demonstrated that TILC2 have tissue-specific effects on PDAC immunity that depended on IL-33/ST2. In fact, pancreatic TILC2, unlike skin TILC2, expressed ST2 and responded to IL-33. In orthotopic PDAC, IL-33/TILC2 axis primed tissue-specific CD8^+^ T cell immunity through recruitment of cross-presenting CD103^+^ DC.

Overall, these studies suggest that despite the divergent effects of ILC2 in tumor immunity, proper activation, such as with IL-33/ST2 stimulation, may promote the anti-tumor functions of these cells through multiple mechanisms, including recruitment of eosinophils and cross-presenting DCs, and tumor cytotoxicity. Given the tissue-specific phenotypes of ILC2, it is possible that such mechanisms may vary depending on the tissue of tumor origin.

## Modulation of immune checkpoints by IL-33

Cancer immunotherapy targeting immune checkpoints has proven effective in treating “hot” tumors through the restoration of preexisting T cell responses. Programmed cell death-1 (PD-1) promotes apoptosis of antigen-specific T-cells, while it sustains regulatory T cell development and function (85, 86). In the TME, up-regulation of PD-1 on T cells occurs in response to activation due to tumor antigens (87), while overexpression of its ligands (PD-L1 and PD-L2) on cancer cells is a well-known immune escape mechanism (88). PD-1 is expressed on a variety of different immune cell types, such as T cells, B cells, NK, myeloid cells, mast cells and innate lymphoid cells (89, 90). Mouse ILC2 express PD-1 in different percentages depending on their tissue of origin and its expression is enhanced by IL-33 stimulation, resulting in impaired Th2-type cytokine production (91, 92). In a mouse model of obesity, TNF-α triggered the expression of IL-33 by pre-adipocytes, which was responsible for PD-1 upregulation on ILC2 (92). Interaction between PD-1^+^ ILC2 and PD-L1^hi^ M1 macrophages resulted in impaired production of IL-5 and IL-13 by ILC2. These findings point to a role of IL-33 in PD-1/PD-L1 pathway.

Emerging data indicate that IL-33 may modulate the PD-1/PD-L1 axis also in cancer. In an AML model, Qin et al. observed that IL-33 induced not only an increase of PD-1 expression on CD8^+^ T cells in peripheral blood, but also higher levels of PD-L1 on tumor cells (32). IL-33 treatment combined with PD-1 blockade prolonged the survival of leukemic mice, providing the first evidence that IL-33 may increase the therapeutic efficacy of immune checkpoint inhibitors. Recently, Moral et al. carried out similar studies on the PDAC mouse model. They showed that IL-33 treatment increased the expression of PD-1 on TILC2, but not in draining LN ILC2, indicating selective activation in the tumor immune compartment (84). Combination of IL-33 and anti-PD-1 reduced tumor growth and improved the survival of PDAC mice in an ILC2-dependent fashion. Of note, this study demonstrated that IL-33 activated TILC2 were direct targets of anti-PD-1. Thus, activation of ILC2s with IL-33 may be a strategy to increase immunotherapy efficacy in ILC2-infiltrated cancers.

IL-33 can affect PD-1/PD-L1 signaling in other immune cells. In a breast cancer model, IL-33 administration increased the percentage of NKp461^+^ PD-1^+^ cells in the TME, while these cells were less frequent in ST2-deficient mice (93). Furthermore, in the B16.OVA melanoma model, systemic administration of IL-33 combined with injection of dectin-1-activated bone marrow-derived DC induced activation and PD-1 expression in OVA-specific CD4^+^ T cells (45). The same group reported that administration of IL-33 reduced the expression of the checkpoint molecules PD-1, LAG-3 and 2B4 on CD8^+^ T cells in mice immunized with “resting” DC (33). Although these two studies suggest that the modulation of immune checkpoints in T cells by IL-33 occurs *via* stimulation of DC, the possibility that IL-33 could also directly activate T cells cannot be excluded. Overall, these findings suggest that IL-33 can affect the PD-1 pathway in several immune cells. Understanding the mechanisms by which IL-33 targets PD-1 in various cancer types may help improving immunotherapy protocols.

The role of IL-33 in the modulation of cytotoxic T-lymphocyte-associated protein 4 (CTLA-4) pathway has been less explored. CTLA-4 is constitutively expressed in regulatory T cells and it is up-regulated in conventional T cells upon activation, where it functions as an inhibitory signal of T cell response (94). In a B16.F1 melanoma pulmonary metastasis model, IL-33 increased the frequency of CD8^+^ T cells expressing PD-1, KLRG-1 and CTLA-4 (95). Hollande et al. reported that tumors expressing high levels of endogenous IL-33 (i.e., Hepa 1-6 and EMT6) respond to combined CTLA-4/PD-1 blockade partially through the help of eosinophils (57). Although this study does not directly address whether IL-33 is relevant for up-regulation of these immune checkpoint molecules, it suggests that local IL-33 and eosinophils recruitment in the TME may promote immunotherapy efficacy. This hypothesis is supported by an increasing number of reports that show a positive correlation between eosinophilia and clinical response to anti-PD-1 and anti-CTLA-4 in cancer patients (47, 96, 97).

## Concluding Remarks

**Figure 1:**
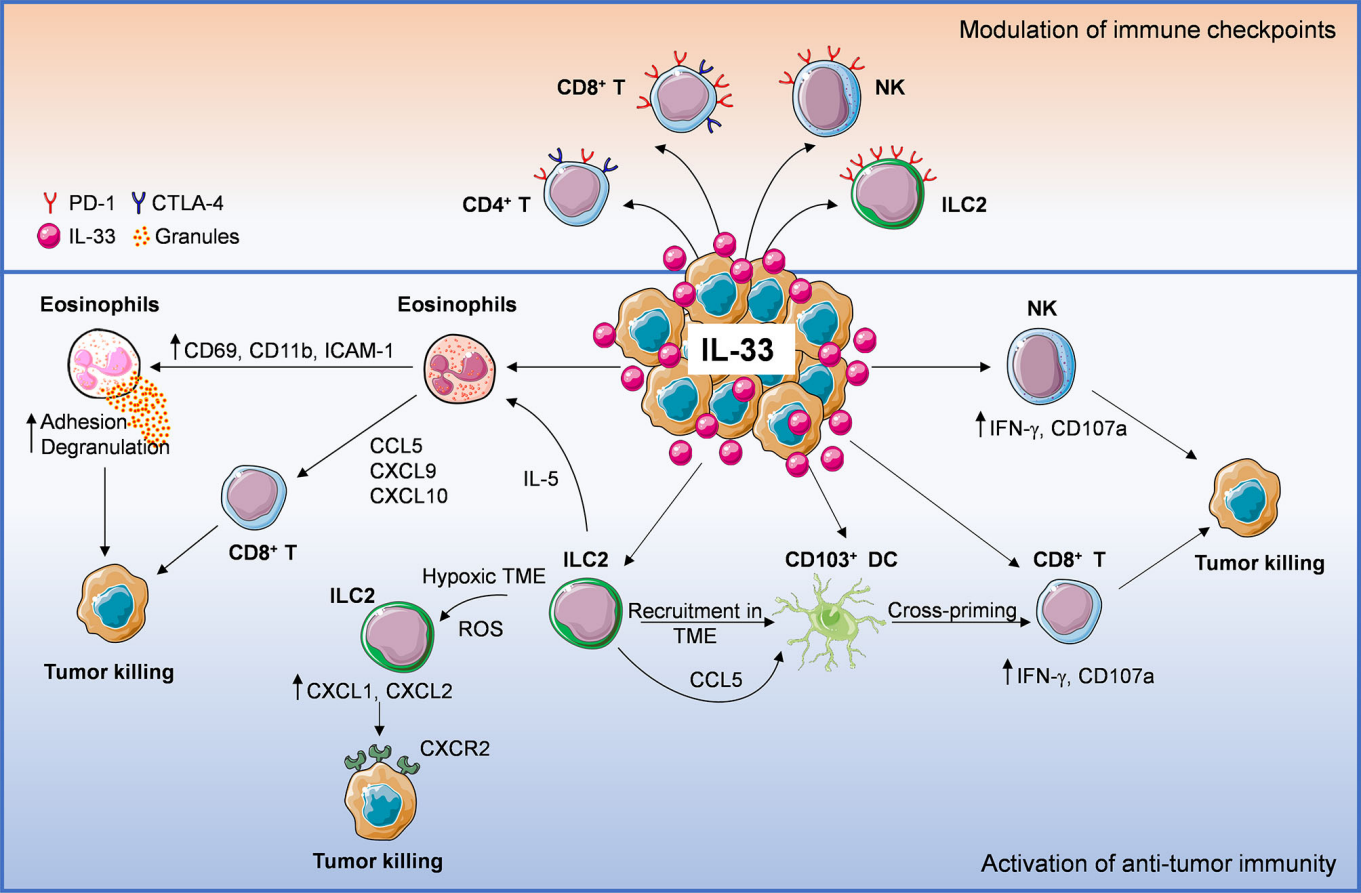
Anti-tumoral mechanisms of interleukin-33 (IL-33) in the tumor microenvironment (TME). IL-33 administration or its physiological expression within the TME leads to direct or indirect recruitment of several immune effector cells such as eosinophils, ILC2, DC, NK cells, CD8^+^, and CD4^+^ T cells, establishing an immune cross-talk or directly controlling tumor growth. ILC2 cells can: 1) directly induce tumor cell killing through CXCL1/CXCL2 release and binding to tumoral CXCR2, 2) promote the recruitment of eosinophils *via* IL-5 production, 3) release CCL5 that facilitates CD103^+^ DC recruitment and cross-priming of CD8^+^ T cells. Following IL-33 exposure, eosinophil recruitment may result in: 1) direct tumor cell killing *via* adhesion-dependent degranulation and 2) release of CD8^+^ T cell-attracting chemokines (CCL5, CXCL9, CXCL10). Moreover, IL-33 can activate NK, CD8^+^ T (directly or *via* stimulation of cross-presenting DC) and CD4^+^ T cells, promoting anti-tumor effector responses. These events may be hindered by concomitant recruitment of ST2^+^ Treg cells. Lastly, IL-33 also up-regulates programmed cell death-1 (PD-1) on T lymphocytes (especially CD8^+^ T), NK cells and ILC2, as well as CTLA-4 on T cells, suggesting that this cytokine may improve the therapeutic response to immune checkpoint inhibitors.

Although the role of IL-33 in cancer immunity remains controversial, it appears that this alarmin has beneficial effects in certain types of experimental tumors, particularly melanoma (16, 20–22, 31, 51, 57). The current literature suggests that the anti-tumor properties of IL-33 are attributable to its capacity to stimulate CD8^+^ T cells, NK, DC, eosinophils and ILC2 (**Figure 1**). Eosinophils are recruited early in the TME and may play a role in the first containment of tumor development (98). A similar function may be potentially played by ILC2, mast cells and basophils. Although relatively rare in human cancers, these cells can release several soluble mediators that may orchestrate tumor immunity in various manners (11, 47, 56, 71, 72, 82). For example, following stimulation with IL-33, eosinophils and ILC2 produce chemokines attracting CD8^+^ T cells (22) and DCs (84), respectively, thus contributing to the initiation of adaptive responses. Furthermore, release of Th2 cytokines, (i.e., IL-4 and IL-5) by basophils, mast cells and ILC2 may promote the recruitment of eosinophils and macrophages that control tumor progression (99, 100). Direct stimulation of NK, CD8^+^ and CD4^+^ T cells by IL-33 has been reported to promote Th1-associated anti-tumor responses in several tumor models (20, 21, 23–26, 28–30). Induction of IL-9 producing CD4^+^ (45) and CD8^+^ (33) T cells by IL-33 may also contribute to anti-tumor immunity. However, IL-33 can induce and amplify Th2 responses in the TME, which may support tumor progression. Moreover, stimulation of ST2^+^ Treg cell recruitment in the TME (3, 16) may further dampen anti-tumor responses. Therefore, tissue-specific environmental factors that shape the local immune TME may dictate the balance of immune responses induced by IL-33. This aspect should be carefully considered when harnessing the IL-33/ST2 axis in tumors particularly enriched in Treg cells, such as breast, lung and gastrointestinal cancers (101).

IL-33 appears to increase the expression of PD-1/PD-L1 and CTLA-4 molecules on certain immune cells (**Figure 1**) and to improve immunotherapy efficacy of checkpoint blockade in some cancer models. The modulation of these and other checkpoint molecules by IL-33 and the immune targets in each cancer type remain to be fully elucidated. In this view, targeting IL-33/ST2 in specific immune cell populations may be a promising strategy to increase the therapeutic response to immune checkpoint inhibitors. Since T_RM_ cells express high levels of immune checkpoint molecules (i.e., PD-1, CTLA-4 and Tim-3), these cells are regarded as key targets of immune checkpoint inhibitors dictating immunotherapy efficacy (102). Future investigation should be directed to evaluate whether targeting the IL-33/ST2 pathway may increase the density of T_RM_ cells in the TME and improve the response to immune checkpoint blockade.

## Author Contributions

All authors contributed to the article and approved the submitted version.

## Funding

This work was supported by grants from AIRC (IG 21366 to GS), CISI-Lab Project (University of Naples Federico II), TIMING Project (Regione Campania) and Campania Bioscience (to SL and GV).

## Conflict of Interest

The authors declare that the research was conducted in the absence of any commercial or financial relationships that could be construed as a potential conflict of interest.

## Abbreviations

AML, acute myeloid leukemia; BMDC, bone marrow-derived DC; CTLA-4, cytotoxic T-lymphocyte-associated protein 4; DC, dendritic cells; HMGB1, high-mobility group box 1 protein; IL-33, Interleukin-33; ILC2, type 2 innate lymphoid cells; LA, lactic acid; LDHA, lactate dehydrogenase A; PDAC, pancreatic ductal adenocarcinomas; PD-1, programmed cell death-1; Tc9, IL-9 producing CD8^+^ T cells; TILC2, tumor-infiltrating ILC2; TME, tumor microenvironment.
